# Altered expression of the suppressors PML and p53 in glioblastoma cells with the antisense-EGF-receptor

**DOI:** 10.1038/sj.bjc.6690798

**Published:** 1999-11

**Authors:** X X Tian, J Y H Chan, J C S Pang, J Chen, J H He, T S S To, S F Leung, H K Ng

**Affiliations:** Departments of Anatomical and Cellular Pathology andClinical Oncology, Sir YK Pao Centre for Cancer, Chinese University of Hong Kong, Prince of Wales Hospital, Shatin, Hong Kong, China; Department of Pathology, Peking Union Medical College Hospital, Beijing, China; Department of Pathology, Tumor Hospital, Sun Yat-Sen University of Medical Sciences, Guangzhou, China; Department of Nursing and Health Sciences, Hong Kong Polytechnic University, Hong Kong, China

**Keywords:** PML, p53, antisense-EGFR, glioblastoma

## Abstract

Gene amplification and enhanced expression of the epidermal growth factor receptor (EGFR) represent the major molecular genetic alteration in glioblastomas and it may play an essential role in cell growth and in the carcinogenic process. On the other hand, the nuclear suppressor proteins PML and p53 are also known to play critical roles in cancer development and in suppressing cell growth. Here we report that, in glioblastoma cells with defective EGFR function, the expressions of both promyelocytic leukaemia (PML) and p53 were altered. Cells that were transfected with the antisense-cDNA of EGFR were found to have more cells in G1 and fewer cells in S phase. In addition, the transfected cells were found to be non-responsive to EGF-induced cell growth. Interestingly, the expression of the suppressors p53 and PML were found to be significantly increased by immunohistochemical assay in the antisense-EGFR cells. Moreover, the PML expression in many of the cells was converted from the nuclear dot pattern into fine-granulated staining pattern. In contrast, the expressions of other cell cycle regulated genes and proto-oncogene, including the cyclin-dependent kinase 4 (cdk4), retinoblastoma, p16^INK4a^ and p21^H-ras^, were not altered. These data indicate that there are specific inductions of PML and p53 proteins which may account for the increase in G1 and growth arrest in antisense-EGFR treated cells. It also indicates that the EGF, p53 and PML transduction pathways were linked and they may constitute an integral part of an altered growth regulatory programme. The interactions and cross-talks of these critical molecules may be very important in regulating cell growth, differentiation and cellular response to treatment in glioblastomas. © 1999 Cancer Research Campaign


*British Journal of Cancer* (1999) **81**(6), 994–1001
© 1999 Cancer Research Campaign
Article no. bjoc.1999.0798

### Altered expression of the suppressors PML and p53 in glioblastoma cells with the antisense-EGF-receptor

**XX Tian[1], JYH Chan[2], JCS Pang[1], J Chen[3], JH He[4], TSS To[5], SF Leung[2] and HK Ng[1]**

Departments of [1]Anatomical and Cellular Pathology and [2]Clinical Oncology, Sir YK Pao Centre for Cancer, Chinese University of Hong Kong, Prince of Wales Hospital, Shatin, Hong Kong, China; [3]Department of Pathology, Peking Union Medical College Hospital, Beijing, China; [4]Department of Pathology, Tumor Hospital, Sun Yat-Sen University of Medical Sciences, Guangzhou, China; [5]Department of Nursing and Health Sciences, Hong Kong Polytechnic University, Hong Kong, China

**Summary** Gene amplification and enhanced expression of the epidermal growth factor receptor (EGFR) represent the major molecular genetic alteration in glioblastomas and it may play an essential role in cell growth and in the carcinogenic process. On the other hand, the nuclear suppressor proteins PML and p53 are also known to play critical roles in cancer development and in suppressing cell growth. Here we report that, in glioblastoma cells with defective EGFR function, the expressions of both promyelocytic leukaemia (PML) and p53 were altered. Cells that were transfected with the antisense-cDNA of EGFR were found to have more cells in G1 and fewer cells in S phase. In addition, the transfected cells were found to be non-responsive to EGF-induced cell growth. Interestingly, the expression of the suppressors p53 and PML were found to be significantly increased by immunohistochemical assay in the antisense-EGFR cells. Moreover, the PML expression in many of the cells was converted from the nuclear dot pattern into fine-granulated staining pattern. In contrast, the expressions of other cell cycle regulated genes and proto-oncogene, including the cyclin-dependent kinase 4 (cdk4), retinoblastoma, $p16^{INK4a}$ and $p21^{H\text{-}ras}$, were not altered. These data indicate that there are specific inductions of PML and p53 proteins which may account for the increase in G1 and growth arrest in antisense-EGFR treated cells. It also indicates that the EGF, p53 and PML transduction pathways were linked and they may constitute an integral part of an altered growth regulatory programme. The interactions and cross-talks of these critical molecules may be very important in regulating cell growth, differentiation and cellular response to treatment in glioblastomas. © 1999 Cancer Research Campaign

**Keywords:** PML; p53; antisense-EGFR; glioblastoma

Brain tumours including the most common form of glioblastomas continue to be important neoplasia in the world affecting a large number of individuals. Recent molecular studies indicate that activation of proto-oncogenes, growth factors and receptors such as the epidermal growth factor receptor (EGFR) was an important event in glioblastomas (Libermann et al, 1985; Nagane et al, 1996; Louis, 1997; Ng and Lam, 1998). EGFR is found to be overexpressed in up to 50% of all reported cases of glioblastomas (Libermann et al, 1985; Wong et al, 1987; Schober et al, 1995). Genetic abnormalities such as gene rearrangement and amplification are detected in the late stages of gliomas, suggesting an important role played by EGFR in tumour proliferation and malignant transformation (Wong et al, 1987). EGFR is a transmembrane glycoprotein of 170 kDa and is composed of an extracellular ligand-binding domain, a single hydrophobic membrane-spanning domain and a cytoplasmic tyrosine kinase domain (Yarden and Ullrich, 1988). Binding of EGF or transforming growth factor-alpha (TGF-α) to EGFR results in receptor dimerization, autophosphorylation of receptor itself and phosphorylation of cellular substrates leading to cell division. The use of antisense RNA or cDNA of EGFR in transfecting tumour cells in order to block the function of enhanced EGFR has been reported previously (Moroni et al, 1992; Chakrabarty et al, 1995; Liu et al, 1995; Wang et al, 1995; Giovanni et al, 1996). However, the effects of antisense-EGFR on the cell cycle-regulated proteins, especially those associated with the nuclear-processing events, are still unclear.

*Received 19 November 1998*
*Revised 24 March 1999*
*Accepted 10 May 1999*

*Correspondence to:* JYH Chan

Moreover, inactivation of tumour suppressor genes has also been documented for the pathogenesis of many neoplastic diseases including the glioblastomas (Louis, 1997). The tumour suppressor p53 is known to play a role in the development of many types of cancers (Smith and Fornace, 1995; Sidransky and Hollstein, 1996), and it is one of the highly mutated genes reported in the literature so far. It plays a pivotal role in cellular response to DNA damage by activating genes for DNA repair, apoptosis, G1 cell cycle arrest and suppression of cell growth (Canman et al, 1994). On the other hand, the *PML* gene (for promyelocytic leukaemia) encodes a growth and transformation suppressor, and has been identified at the non-random chromosomal translocation break point of acute promyelocytic leukaemia (APL) (Chang et al, 1995; Lamond and Earnshaw, 1998). PML is a nuclear phosphoprotein that has been shown to inhibit cell growth and the transformed phenotypes of tumour cells (Mu et al, 1994; Chang et al, 1995; Liu et al, 1995) and is localized in nuclear bodies (NB) or PML oncogenic domain (POD) (Koken et al, 1995). Moreover, PML is involved in cell cycle arrest and viral replication (Everett and Maul, 1994; Pivion et al, 1995), and significant increase in PML nuclear bodies and a non-aggregated soluble form were found in cells progress through G1 and S phase of the cell cycle (Terris et al, 1995).

We have recently reported on the use of antisense-EGFR transfection to inhibit the proliferation and to induce the differentiation of glioblastoma cells (Tian et al, 1998). We have also documented

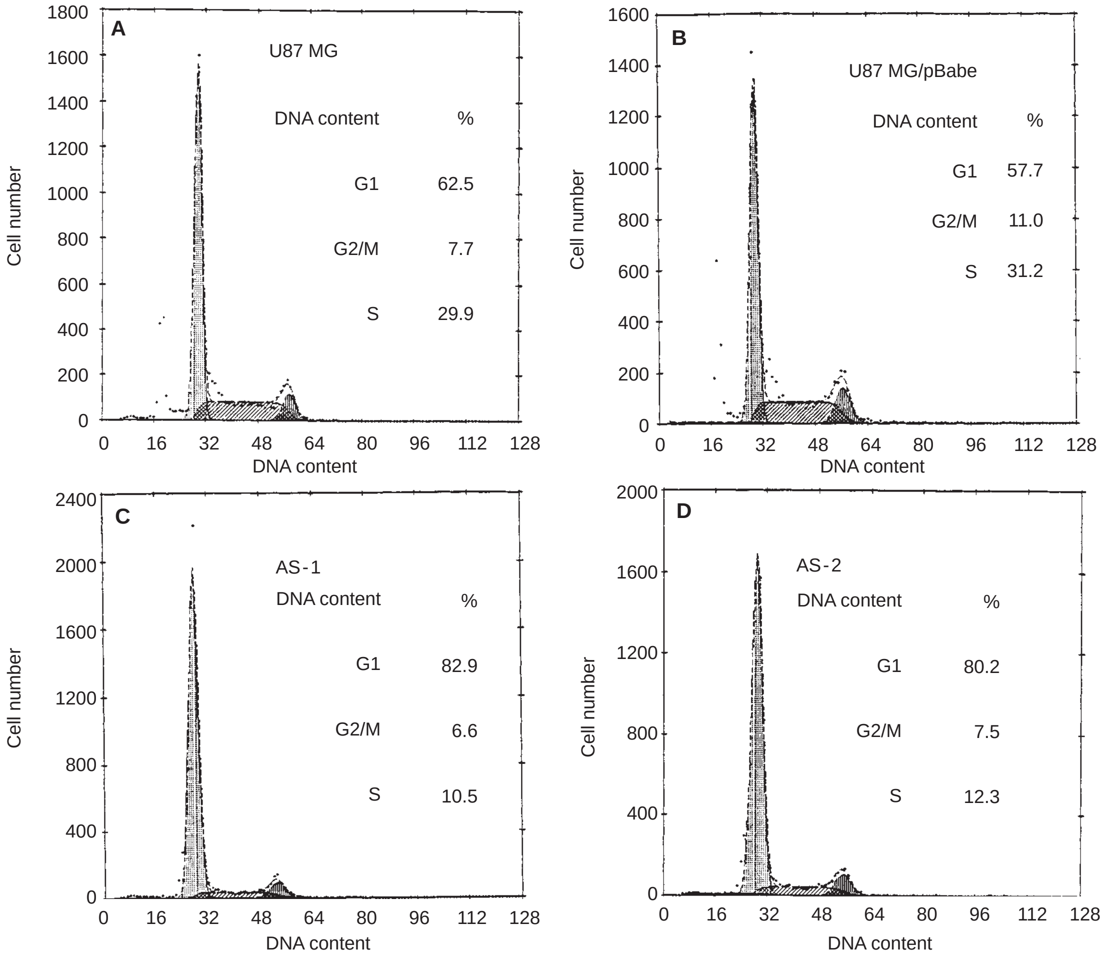

**Figure 1** Flow cytometry analysis of glioblastoma cells transfected with the antisense-EGFR. U87MG cells were transfected with the vector alone or with the antisense-EGFR constructs as described in Methods. The stable clones were isolated and analysed by flow cytometry for different phases of the cell cycle the cell cycle regulation of DNA damage-induced expression of the suppressor PML, and that p53 may regulate the expression of PML (Chan et al, 1997). Moreover, altered expression of PML in hepatocellular carcinomas was also identified (Chan et al, 1998). To determine if the nuclear processing events may be involved in the membrane signal-transduction pathway of EGFR, we analysed the expression of several cell cycle-regulated proteins, including p53 and PML, in human glioblastoma cells treated with the antisense-EGFR.

### MATERIALS AND METHODS

### Construction of antisense-EGFR vector

The construction of the antisense-EGFR and the cloning of the transfected cells have been described previously (Tian et al, 1998). The EGFR cDNA fragment (*Bam*HI-*Bam*HI) derived from *PE*7 was inserted in reverse orientation at the *Bam*HI site of the *pBabe-puro* vector (Morgenstern and Land, 1990; Liu et al, 1995). This cDNA corresponds to the last 256 amino acid residues of the extracellular domain, the entire transmembrane domain and the first 61 amino acid residues of the cytoplasmic domain of EGFR.

### Cell culture and transfection

The human glioblastoma cell line U87MG (American Type Culture Collection, Rockville, MD, USA) was grown in minimum essential medium-alpha (MEM-α) medium (Gibco, Grand Island, NY, USA) supplemented with 10% fetal bovine serum (FBS), 100 μg $ml^{-1}$ streptomycin and 100 U $ml^{-1}$ penicillin, in a humidified atmosphere of 5% carbon dioxide at 37°C. Cells were transfected with the antisense-EGFR constructs using the Transfectam reagent (Promega Corp., Madison, WI, USA). Stable clones (AS-1 and AS-2) were selected in 1 μg $ml^{-1}$ puromycin (Sigma Chemical Co., St Louis, MO, USA) as described previously (Tian et al, 1998).

The effect of EGF was determined by incubating cells with media with low concentration of FBS (1%). Approximately $1 \times 10^4$ glioma cells were incubated in MEM-α supplemented with 10% FBS for 24 h at 37°C. Cells were then treated with 100 ng $ml^{-1}$ of EGF in MEM-α +1% FBS. The viability of the cells was determined by the trypan blue dye exclusion method, and all the experiments of cell growth were performed in triplicates each for three times.

© 1999 Cancer Research Campaign

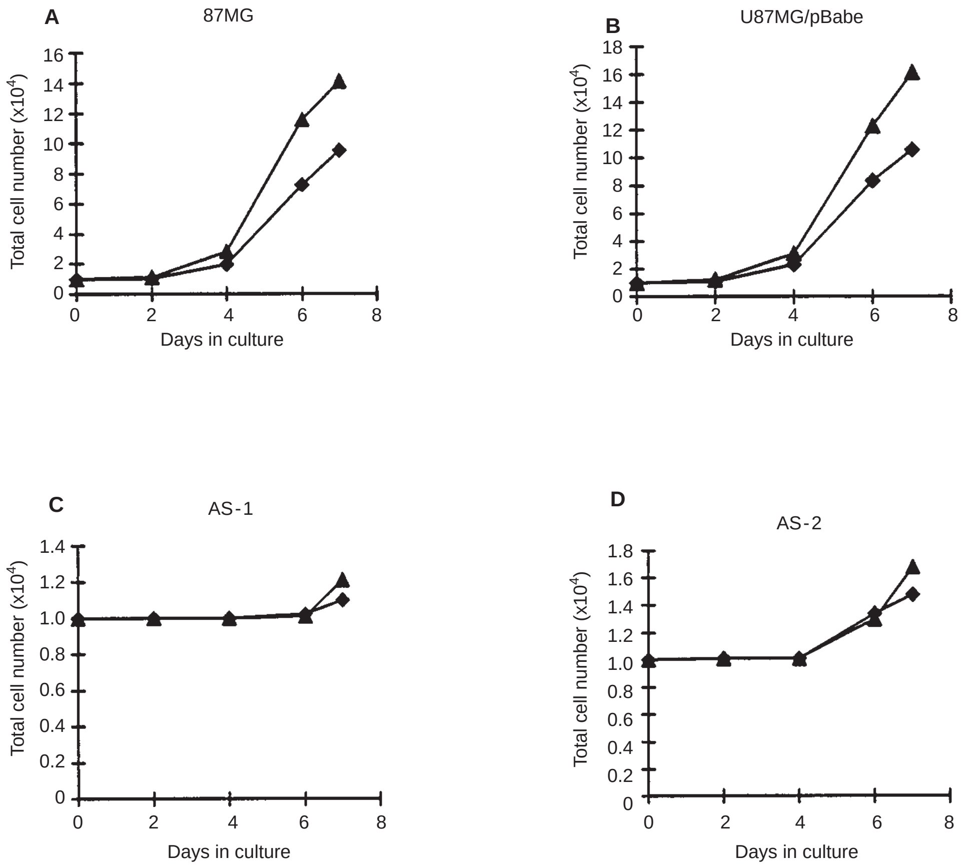

**Figure 2** Effect of EGF on glioblastoma cells transfected with the antisense-EGFR. U87G cells, cells transfected with the empty vector *pBabe-puro* or the antisense-EGFR constructs were resuspended in media containing low level of FBS (1%). EGF was added to the cells at 0.1 µg $ml^{-1}$, and the rate of cell growth was determined at various time intervals. (**A**) Parental cells; (**B**) cells treated with empty vector; (**C** and **D**) antisense-EGFR clones AS-1 and AS-2 respectively. The symbol (▲) represents cells treated with EGF, while the symbol (◆) represents untreated cells

### Flow cytometry analysis of DNA ploidy

The method for measuring the proportions of cells in the various cell cycle phases was performed with a Coulter Epics XL flow analyser (Coulter Corp., FL, USA) as described previously (Tian et al, 1998). In brief, cells were fixed in ethanol, stained with 50 µg $ml^{-1}$ propidium iodide (Sigma) containing 0.1% Triton X-100 and 1% RNAase, and analysed by the flow analyser. The percentages of cells in different phases of the cell cycle were analysed by the Multicycle DNA Cell Cycle program (Phoenix Laboratories, San Diego, CA, USA).

### Immunohistochemical staining

Immunohistochemical staining was performed with the following antibodies: affinity-purified rabbit antibody raised against the recombinant PML protein (Chan et al, 1997), rabbit anti-human EGFR antibody (Sc-03, Santa Cruz Biotechnology, Inc., Santa Cruz, CA, USA), rabbit anti-human cyclin-dependent kinase 4 (cdk4) (Sc-601, Santa Cruz Inc.), mouse anti-human Rb (14001A, PharMingen, San Diego, CA, USA), mouse anti-human p16 (13251A, PharMingen), mouse anti-human $p21^{H\text{-}ras}$ (M637, DAKO A/S, Copenhagen, Denmark), or p53 (DO7, Novocastra Lab., Newcastle upon Tyne, UK). Cells were cultured on cover-slip, and fixed in cold methanol. The slides were boiled in 10 mM sodium citrate buffer (pH 6.8) for 10 min in a microwave oven. After treatment with 3% hydrogen peroxide in methanol, the samples were blocked with the blocking solution (5% bovine serum albumin (BSA) in phosphate-buffered saline (PBS)), and then incubated with the first antibodies against: PML (1:2000), EGFR (1:50), cdk4 (1:25), retinoblastoma (Rb) (1:50), p16 (1:25), p53 (1:50) or H-*ras* (1:25), for 2 h at room temperature. After washing, the samples were incubated for 30 min with the secondary antibodies, and were visualized by the peroxidase anti-peroxidase method or avidin biotin–peroxidase complex method. After counterstaining weakly with haematoxylin and briefly washing with 30 mM ammonium hydroxide, the slides were mounted. All of the immunostaining was repeated at least twice.

### RESULTS

### Decrease in S phase fraction of glioblastoma cells with antisense-EGFR

The antisense-EGFR constructs were transfected into a glioblastoma cell line U87MG and stable transfected clones were isolated. Flow cytometry analysis of the stable antisense-EGFR clones in U87MG cells indicated that the parental cell U87MG contained 62.5% G1 cells while the G2/M was 7.7%, and S phase cells was 29.9% as shown in Figure 1A. Similar ratio was obtained in

 © 1999 Cancer Research Campaign

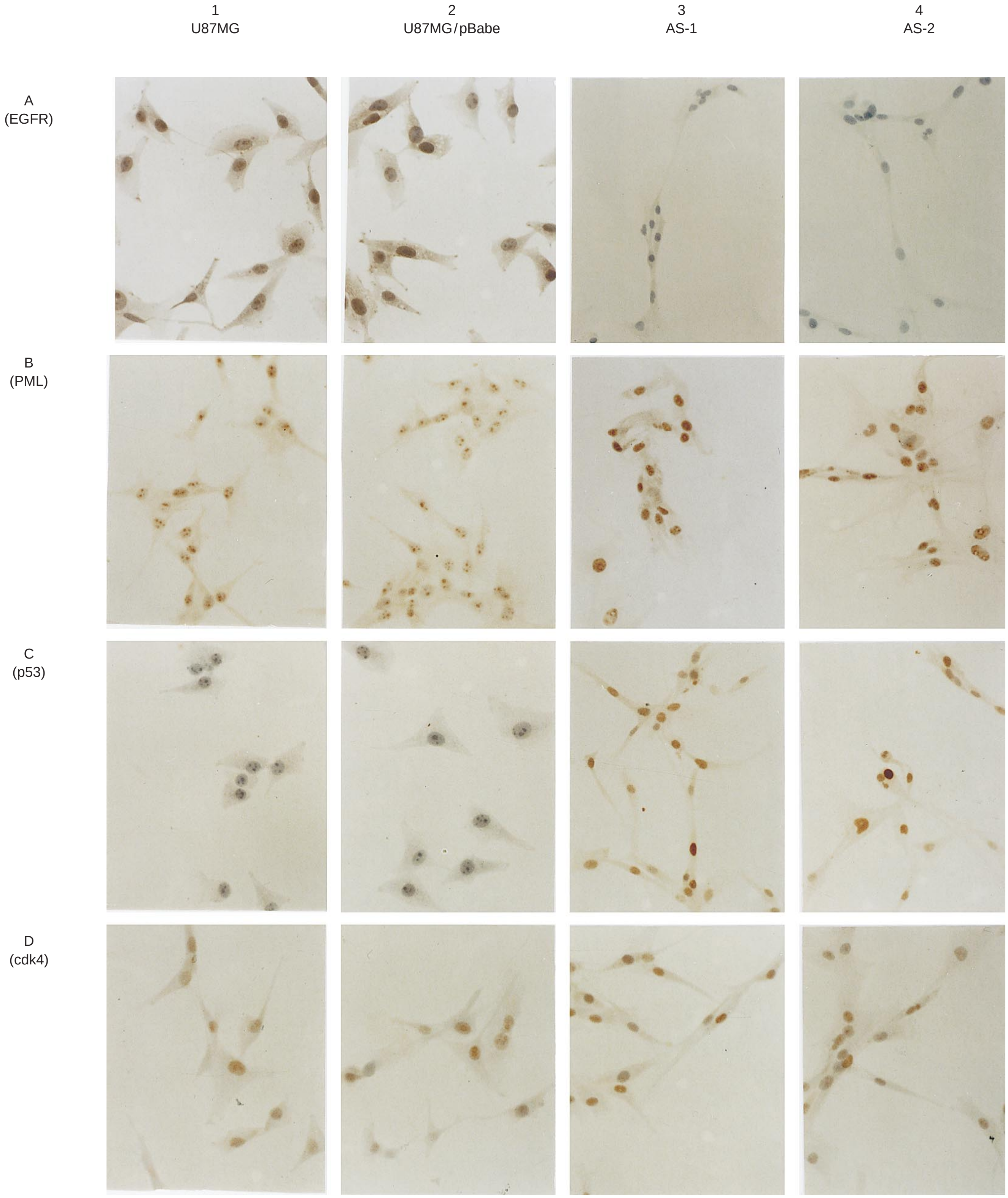

**Figure 3** Altered expression of EGFR, PML, p53 and cdk4 in glioblastoma cells transfected with the antisense-EGFR constructs. The parental cells U87MG (column 1) and cells with the empty vector *pBabe-puro* (column 2) or with the antisense-EGFR (AS-1 and AS-2) in columns 3 and 4 respectively were immunohistochemically stained with antibodies against EGFR (row A), PML (row B), p53 (row C), and Cdk-4 (row D) respectively as described in Methods. The samples stained with PML in row B were not counter-stained and the original magnification for all of the prints was 200×

*© 1999 Cancer Research Campaign*

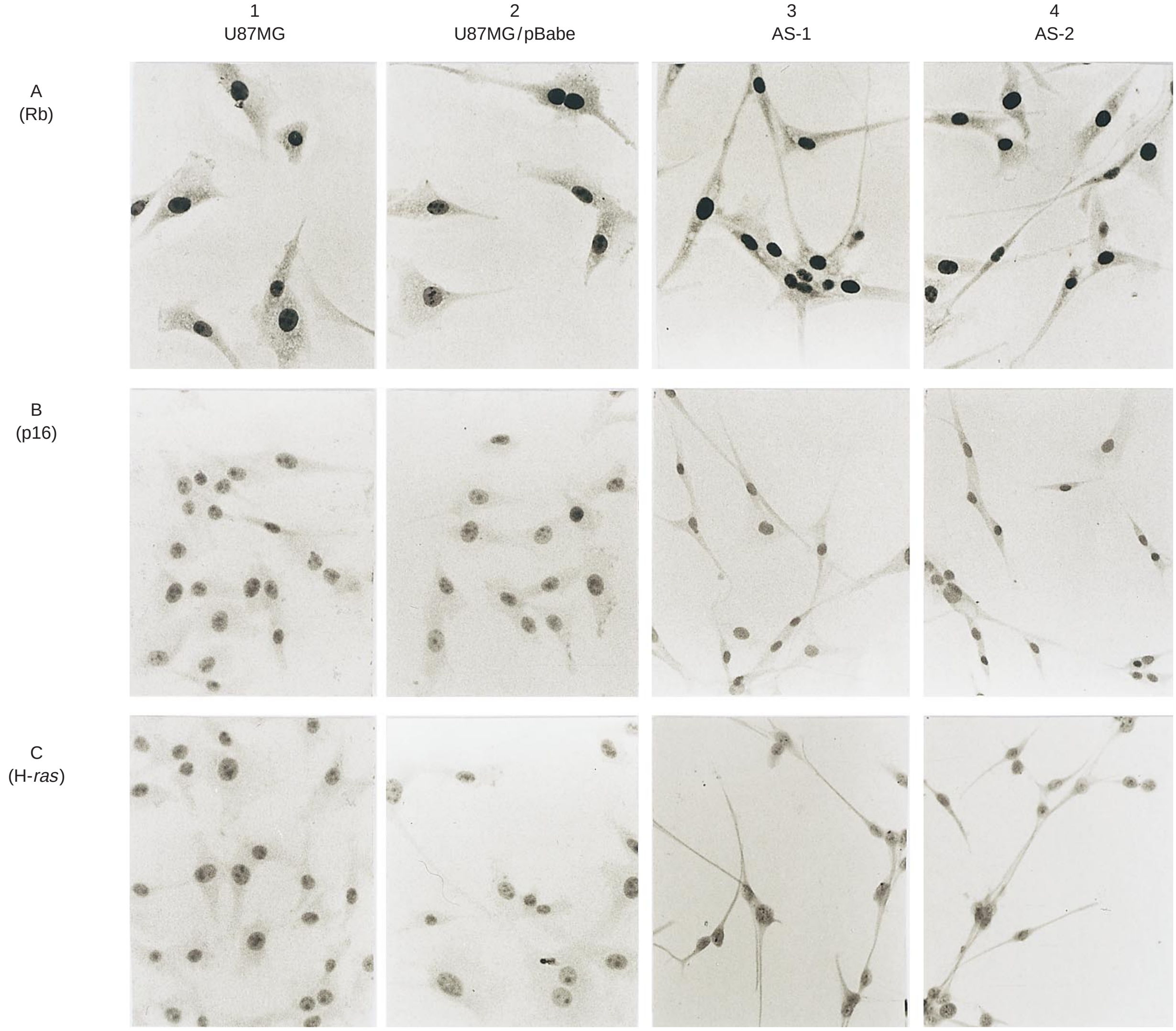

**Figure 4** Expression of Rb, p16 and H-*ras* in glioblastoma cells transfected with the antisense-EGFR. U87MG cells, cells with the empty vector or antisense-EGFR were immunohistochemically stained with a rabbit polyclonal antibody against the Rb (row A), p16 (row B) and H-*ras* (row C) respectively as described in Methods. Column 1, parental U87MG cells; column 2, cells with the vector *pBabe-puro*; columns 3 and 4, antisense-EGFR clones AS-1 and AS-2 respectively. The original magnification for all of the prints was 200×

U87MG cells transfected with the vector *pBabe-puro* alone (U87MG/pBabe) (Figure 1B). However, in cells transfected with the antisense-EGFR constructs, the fraction of G1 cells increased to 82.9% and 80.2% in two separate clones, AS-1 (Figure 1C) and AS-2 (Figure 1D) respectively. The ratio of G2/M cells did not change much, at approximately 6.6–7.5%, but the S phase cells decreased to 10.5% and 12.3% respectively for AS-1 and AS-2 cells. The experiment was repeated three times and similar results were obtained.

### Effect of EGF on glioblastoma cells with antisense-EGFR

To determine if the transfected cells would still respond to the proliferative effect of EGF, cells were resuspended in media with low level of FBS (1%), and EGF was added to the media of different cell clones. The growth rates of these cells were then monitored. As shown in Figure 2A and 2B for the control parental U87MG cells and the cells with the empty vector *pBabe-puro* respectively, the cells responded positively to the growth-stimulation of EGF. In contrast, the clones treated with antisense-EGFR (AS-1 and AS-2 for Figure 2C and 2D respectively), did not respond well to the exogenously added EGF indicating that the antisense-EGFR abrogated the ability of the cells to respond to EGF. At 7 days post-treatment with EGF, the cell numbers for the parental and the empty vector were 14.1 and 16.2 ($\times 10^4$) respectively, while the numbers for the two antisense EGFR-clones, AS-1 and AS-2, were 1.2 and 1.7 ($\times 10^4$) respectively.

### Suppression of EGFR expression in glioblastoma cells treated with antisense-EGFR

The effect of the antisense-EGFR on the expression of EGFR was then determined. As shown in Figure 3 row A, the expression of EGFR was analysed by immunohistochemical staining with an antibody against EGFR. Strong staining of EGFR was found in the

 *© 1999 Cancer Research Campaign*

parental U87MG cells and the vector-transfected cells U87MG/pBabe. In contrast, weak or undetectable staining was found in the antisense-EGFR cells AS-1 and AS-2 respectively. There were significant decreases in EGFR expression in the antisense-EGFR clones as compared to the controls. We have previously shown by Western blotting (Tian et al, 1998) that the EGFR protein in the two antisense clones (AS-1 and AS-2) were undetectable. However, the control cells expressed a large amount of EGFR protein. In addition, the parental U87MG and the vector alone-transfected clone were large, round or spindle-shape cells which are characteristics of transformed cells, while the antisense clones AS-1 and AS-2 were small, elongated and bipolar in shape with long processes, which are properties of differentiated glial cells. Moreover, the parental and empty vector-transfected cells had very low levels of expression of the differentiation marker GFAP, while the AS-1 and AS-2 contained high GFAP protein (Tian et al, 1998).

### Altered expression of PML in glioblastoma cells with the antisense-EGFR

We have previously published on the suppressor *PML* being a cell cycle-regulated gene (Chan et al, 1997) and that transfection of *PML* arrests cells in G1. In addition, the optimal expression of PML is also at G1 (Mu et al, 1995). Thus, we were interested to see whether the antisense-EGFR would alter the expression of cell cycle-regulated genes such as the *PML*. Immunohistochemical staining of PML was performed on control cells and cells treated with antisense-EGFR. As shown in Figure 3 row B, PML expression in 80–90% of the parental cells and the vector-alone cells, displayed a nuclear dot like pattern. The number of POD was about 2–6 per cell and the average number of dots was 4 per cell. Moreover, the intensity of dots in the majority of cells was relatively weak. In contrast, increased PML labelling was found in the antisense clones (AS-1 and AS-2 in Figure 3, panels B3 and B4 respectively). The intensity was strong and the number of nuclear bodies increased to 10 or more. Around 60–70% of the antisense-EGFR-treated cells showed a patched-like pattern or a homogeneous granulated pattern of strong PML staining, consistent with data reported previously by us and by others in cell cycle-arrested cells (Koken et al, 1995; Chan et al, 1997). These results indicate that enhanced expression of PML and possibly a redistribution of PML were found in antisense-EGFR cells as compared to the control cells.

### Enhanced expression of p53 in glioblastoma cells with the antisense-EGFR

Since it is known that the tumour suppressor p53 plays a pivotal role in inhibiting cell growth and in G1-cell cycle arrest, and that p53 may regulate the expression of PML or vice versa, we were interested to know if the expression of p53 was also altered in the cells treated with antisense-EGFR. It was reported previously that p53 in U87MG is wild-type (Gomez-Manzano et al, 1997). We then determined the expression of p53 with an antibody against p53 in these four different clones as shown in Figure 3, row C. Immunohistochemical staining with an antibody against p53 (DO-7) labelled a large number of the cells negative or weak staining in the parental cells and the empty vector-transfected cells as shown in Figure 3, Panels C1 and C2 respectively. In contrast, the p53 staining of a considerable number of cells in the antisense-EGFR clones, AS-1 and AS-2, was much stronger (Figure 3, panels C3 and C4 respectively). Approximately 1000 cells were counted for each slide of three independent experiments, and the percentage of cells with strong staining of p53 in parental and empty vector were calculated to be 3% ± 2 standard deviation (s.d.) and 3% ± 3 (s.d.), while the AS-1 and AS-2 cells were 11% ± 5 (s.d.) and 8% ± 3 (s.d.) respectively. The proportion of cells with intermediate levels of p53 staining were 18% ± 4 (s.d.), 14% ± 2 (s.d.), 54% ± 3 (s.d.) and 50% ± 7 (s.d.) for parental, empty vector, AS-1 and AS-2 respectively. In contrast, the proportions of cells stained negative or weak were 79% ± 6 (s.d.), 83% ± 4 (s.d.), 35% ± 3 (s.d.) and 41% ± 5 (s.d.) for parental, empty-vector, AS-1 and AS-2 respectively. About three-fold increase in the strong staining cells and intermediate staining cells, and twofold decrease of cells stained weak or negative were observed in the antisense-EGFR clones, which indicate that p53 is involved in the EGFR pathway also.

### Expression of cdk4, Rb, p16$^{INK4A}$, and H-*ras* in

To determine if other cell cycle-regulated gene products were also altered in the antisense-EGFR-transfected cells, cdk4 was stained in control cells and antisense clones. As shown in Figure 3, row D, the protein of cdk4 was mostly found in the nucleus of U87MG and the U87MG/pBabe cells (Figure 3, panels D1 and D2 respectively) similar to those of PML and p53. Unlike p53 and PML, the staining of cdk4 in antisense-EGFR clones of AS-1 and AS-2 (Figure 3, panels D3 and D4 respectively) were similar to the controls.

Similarly, we have examined the expression of another tumour suppressor and cell cycle regulator, the retinoblastoma gene (Rb) in these cells. As shown in Figure 4, row A, the expression of Rb was analysed by immunohistochemical staining with an anti-Rb antibody. Rb staining was found to be quite strong and confined to the nucleus. However, no detectable changes in Rb expression were found in the AS-1 and AS-2 cells (Figure 4, panels A3 and A4 respectively) as compared to the parental cells, and vector-transfected cells (Figure 4, panels A1 and A2 respectively).

Similarly for another tumour suppressor protein p16$^{INK4a}$ which is known to regulate cell cycle progression and arrests cells by inhibiting the activity of cdk. However, the staining of p16 in these cells was found to be very weak or negative in all of the four clones (Figure 4, row B).

For the H-*ras* proto-oncogene (Figure 4, row C), no gross alterations in the expression of p21-H-*ras* protein were found in the four different clones, and most of the cells showed negative or weak staining in the cytoplasm.

### DISCUSSION

In this study, a glioblastoma cell line U87MG that was previously shown to have over-expressed EGFR and with wild-type p53, was transfected with the antisense-EGFR constructs. The stable transfectants were found to be suppressed in cell growth, increased in G1 arrest and decreased in Ki-67 staining (Tian et al, 1998) which are in accordance with the notion that enhanced expression of EGFR are, at least in part, responsible for the aggressive cell growth associated with the tumour cells. This is the first report showing that the enhanced expressions of the nuclear suppressors

© 1999 Cancer Research Campaign 

p53 and PML were associated with the increased G1 cells in the antisense-EGFR transfectants. These results are also in keeping with the role of PML and p53 as growth suppressors and cell cycle G1 regulators. Increased expression of PML and wild-type p53 protein has been shown to be associated with G1 arrest and inhibition of cell growth (Mu et al, 1994; Chan et al, 1998). It may indicate that p53 and PML are in the EGFR mitogen-activation cascade and they are the downstream targets of EGFR. However, the expressions of other G1 phase regulatory proteins including cdk4, Rb and p16$^{INK4a}$, as well as the proto-oncoprotein H-*ras* were not altered in the antisense-EGFR clones. Thus, the altered expressions of p53 and PML in the blocked EGFR pathway appear to be specific in these glioma cells. Moreover, there are reports on the nuclear localization of EGFR (Cao et al, 1995; Bandyopadhyay et al, 1998) which raise the possibility that EFGR might regulate PML or p53 directly.

However, a report on the elevation of EGFR leading to increased resistance to cisplatin induced cytotoxicity had been documented (Dickstein et al, 1995). In addition, by blocking EGFR with anti-EGFR antibody, cells were found to be more sensitive to cisplatin treatment (Mendelsohn and Fan, 1997). We have analysed apoptosis induced by cisplatin in U87MG cells with the TUNEL assay. Preliminary data revealed that 12~15% of the cells were positive in AS-1 and AS-2 respectively, while only 7~9% were found in the parental and empty vector-transfected cells (Tian et al, unpublished data). These results apparently are in keeping with the role of p53 and PML as apoptosis regulators (Gomez-Manzano et al, 1997; Hess and Korsmeyer, 1998). However, the antisense-RNA of EGFR was found to block cisplatin-induced programmed cell death in a human breast cancer cell line MDA-468 in one report which differed from ours and others (Dixit et al, 1997). The reason for this discrepancy is currently unknown, but the differential molecular profiles of individual cell lines may account for the differential effect (Mendelsohn and Fan, 1997). For example, MDA-468 contains mutated p53 and the Rb gene is deleted (Mendelson and Fan, 1997), while our cell line U87MG contains wild-type p53 and the Rb gene (Gomez-Manzano et al, 1997).

On the other hand, the PML expression was found to convert from the nuclear dot pattern into patched-like pattern or as fine-granulated homogeneous staining pattern in cells treated with the antisense-EGFR. Enhanced PML protein was associated with inflammation, and altered expression was found during the oncogenesis of many types of cancers (Koken et al, 1995; Terris et al, 1995). We have previously shown that the optimal expression of PML in the POD structure is at G1 arrest (Chan et al, 1997). PML co-localized with proteins including Sp-100, NDP55, PIC 1, Int-6, and SUMO in the POD structure (Lamond and Earnshaw, 1998), and may play a role in viral infection and replication (Everett and Maul, 1994; Puvion-Dutilleul et al, 1995). One hypothesis put forth (Maul et al, 1995) is that the nuclear bodies or POD may represent storage sites of certain matrix proteins readily accessible throughout the chromatin in response to stress or other effectors that induce global nuclear changes. Thus, PML may act as a matchmaker to recruit or sequester other proteins in or out of the POD. The redistribution of PML from a few dotted structures to granular staining pattern in antisense-EGFR cells may represent global rearrangements of nuclear proteins.

The relationship of EGFR and p53 in brain tumours has been reported previously which includes a report on the immunohistochemical staining of astrocytomas (Kordek et al, 1995). A small percentage of samples (9%) was stained double-positive for p53 and EGFR. However, these cases did not show any difference in survival rate nor in the tumour grades. Since the enhanced expression of p53 may indicate the presence of a mutated protein or increased expression of normal protein, the significance of these findings is not known. Another report showed that p53 could activate the trk tyrosine kinase by hyper-phosphorylation, but had no effect on EGFR (Montano, 1997), while EGFR can be activated by p53 through the binding to a p53 response element at the EGFR promoter (Ded et al, 1994; Ludes-Meyers et al, 1996). Our data provide strong evidence for the reverse pathway of EGFR in regulating p53, indicating that the interaction of these molecules were linked. On the other hand, another report (Vallian et al, 1997) suggested that PML, as a transcriptional regulator, might repress the transcriptional activity of the EGFR promoter also. Thus, the regulation of membrane signals and nuclear events appears to be quite complex (Tang et al, 1997). Nevertheless, the autocrine loop of TGF-α and EGF/EGFR plays a dominant role in the aberrant cell growth of many types of tumours including glioblastomas. The elucidation of the molecular details of this signal transduction pathway especially the nuclear events is, therefore, crucial in developing effective therapeutic strategy for these tumors.

### ACKNOWLEDGEMENTS

This research was supported in part by CUHK-UGC Grants 026809335 and 220404940, RGC Earmarked Grants CUHK 4280/98 M (JYH Chan) and CUHK4260/97 M (SF Leung).

### REFERENCES

Bandyopadhyay D, Maddal M, Adam L, Mendelsohn J and Kumar R (1998) Physical interaction between epidermal growth factor receptor and DNA-dependent protein kinase in mammalian cells. *J Biol Chem* **273**: 1568–1573

Canman CE, Chen CY, Lee MH and Kastan MB (1994) DNA damage responses: p53 induction, cell cycle perturbations and apoptosis. *Cold Spring Harb Sym Quan Biol* **LIX**: 277–286

Cao H, Lei ZM, Bian L and Rao CV (1995) Functional nuclear epidermal growth factor receptors in human choriocarcinoma JEG-3 cells and normal human placenta. *Endocrinology* **136**: 3163–3172

Chakrabarty S, Rajagopal S and Huang S (1995) Expression of antisense epidermal growth factor receptor RNA downregulates the malignant behavior of human colon cancer cells. *Clin Exp Metastasis* **13**: 191–195

Chan JYH, Li L, Fan YH, Mu ZM, Zhang WW and Chang KS (1997) Cell-cycle regulation of DNA damage-induced expression of the suppressor gene *PML*. *Biochem Biophys Res Commun* **240**: 640–646

Chan JYH, Chin W, Liew CT, Chang KS and Johnson PJ (1998) Altered expression of the growth- and transformation-suppressor PML in human hepatocellular carcinomas and in hepatitis tissues. *Eur J Cancer* **34**: 1015–1022

Chang KS, Fan YH, Andreeff M, Liu J and Mu ZM (1995) The PML gene encodes a phosphoprotein associated with the nuclear matrix. *Blood* **85**: 3646–3653

De Giovanni C, Landuzzi L, Frabetti F, Nicoletti G, Griffoni C, Rossi I, Mazzotti M, Scotto L, Nanni P and Lollini PL (1996) Antisense epidermal growth factor receptor transfection impairs the proliferative ability of human rhabdomyosarcoma cells. *Cancer Res* **56**: 3898–3901

Ded SP, Munoz RM, Brown DR, Subler MA and Sumitra D (1994) Wild-type human p53 activates the human epidermal growth factor receptor promoter. *Oncogene* **9**: 1341–1349

Dixit M, Yang JL, Poirier MC, Price JO, Andrews PA and Arteaga CL (1997) Abrogation of cisplatin-induced programmed cell death in human breast cancer cells by epidermal growth factor antisense RNA. *J Natl Cancer Inst* **89**: 365–373

Everett RD and Maul GG (1994) HSV-1 IE protein Vmw110 causes redistribution of PML. *EMBO J* **13**: 5062–5069

Gomez-Manzano C, Fueyo J, Kyritsis AP, McDonnell TJ, Steck PA, Levin VA and Yung WK (1997) Characterization of p53 and p21 functional interactions in glioma cells en route to apoptosis. *J Natl Cancer Inst* **89**: 1036–1044

*© 1999 Cancer Research Campaign*

Hartwell LH and Kastan MB (1994) Cell cycle control and cancer. *Science* **266**: 1821–1828

Koken MHM, Linares-Cruz G, Quignon F, Viron A, Chelbi-Alix MK, Sobczak-Thepot J, Juhlin L, Degos L, Calvo F and de The H (1995) The PML growth-suppressor has an altered expression in human oncogenesis. *Oncogene* **10**: 1315–1324

Kordek R, Biernat W, Alwasiak ZJ, Maculewicz R, Yanagihara R and Liberski P (1995) p53 protein and epidermal growth factor receptor expression in human astrocytomas. *J Neuro-Oncol* **26**: 11–16

Lamond AI and Earnshaw WC (1998) Structure and function in the nucleus. *Science* **280**: 547–553

Libermann TA, Nusbaum HR, Razon N, Kris R, Lax I, Soreq H, Whittle N, Waterfield MD, Ullrich A and Schlessinger J (1985) Amplification, enhanced expression and possible rearrangement of EGF receptor gene in primary human brain tumors of glial origin. *Nature* **313**: 144–147

Liu JH, Mu ZM and Chang KS (1995) PML suppresses oncogenic transformation of NIH-3T3 cells by activated *neu*. *J Exp Med* **181**: 1965–1973

Liu T, Chen J and Zeng C (1995) Effects of antisense epidermal growth factor and its receptor retroviral expression vectors on cell growth of human pancreatic carcinoma cell line. *Chin Med J* (Engl) **108**: 653–659

Louis DN (1997) A molecular genetic model of astrocytoma. *Brain Pathol* **7**: 755–764

Ludes-Meyers JH, Subler MA, Shivakumar CV, Munoz RM, Jiang P, Bigger JE, Brown DR, Deb SP and Deb S (1996) Transcriptional activation of the human epidermal growth factor receptor promoter by human p53. *Mol Cell Biol* **16**: 6009–6019

Maul GG, Yu E, Ishov AM and Epstein AL (1995) Nuclear domain 10 (ND10) associated proteins are also present in nuclear bodies and redistribute to hundreds of nuclear sites after stress. *J Cell Biochem* **59**: 498–513

Mendelsohn J and Fan Z (1997) Epidermal growth factor receptor family and chemosensitization. *J Natl Cancer Inst* **89**: 341–343

Montano X (1997) p53 associates with trk tyrosine kinase. *Oncogene* **15**: 245–256

Morgenstern JP and Land H (1990) Advanced mammalian gene transfer: high titre retroviral vectors with multiple drug selection markers and a complementary helper-free packaging cell line. *Nucleic Acid Res* **18**: 3587–3596

Moroni MC, Willingham MC and Beguinot L (1992) EGF-R antisense RNA blocks expression of the epidermal growth factor receptor and suppresses the transforming phenotype of a human carcinoma cell line. *J Biol Chem* **267**: 2714–2722

Mu ZM, Chin KV, Liu JH, Lozano G and Chang KS (1994) PML, a growth suppressor disrupted in acute promyelocytic leukemia. *Mol Cell Biol* **14**: 6858–6867

Nagane M, Coufal F, Lin H, Bogler O, Cavenee WK and Huang HJS (1996) A common mutant epidermal growth factor receptor confers enhanced tumorigenicity on human glioblastoma cells by increasing proliferation and reducing apoptosis. *Cancer Res* **56**: 5079–5086

Ng HK and Lam PYP (1998) The molecular genetics of central nervous system tumours. *Pathology* **30**: 196–202

Puvion-Dutilleul F, Chelbi-Alix M, Koken M, Quignon F, Puvion E and de The H (1995) Adenovirus infection induces rearrangements in the intranuclear distribution of the nuclear body-associated PML protein. *Exp Cell Res* **218**: 9–16

Schober R, Bilzer T, Waha A, Reifenberger G, Wechsler W, von Deimling A, Wiestler OD, Westphal M, Kemshead JT and Vega F (1995) The epidermal growth factor receptor in glioblastoma: genomic amplification, protein expression, and patient survival data in a therapeutic trial. *Clin Neuropathol* **14**: 169–174

Sidransky D and Hollstein M (1996) Clinical implications of the p53 gene. *Annu Rev Med* **47**: 285–301

Smith ML and Fornace AJ (1995) Genomic instability and the role of p53 mutations in cancer cells. *Curr Opin Oncol* **7**: 69–75

Tang P, Steck PA and Yung WK (1997) The autocrine loop of TGF-alpha/EGFR and brain tumors. *J Neuro-Oncol* **35**: 303–314

Terris B, Baldin V, Dubios S, Degott C, Flejou JF, Henin D and Dejean A (1995) PML nuclear bodies are general targets for inflammation and cell proliferation. *Cancer Res* **55**: 1590–1597

Tian XX, Lam PYP, Chan J, Pang JCS, To SST, Di-Tomaso E and Ng HK (1998) Antisense EGF receptor RNA transfection in human malignant glioma cells leads to inhibition of proliferation and induction of differentiation. *Neuropath Appl Neurobiol* **24**: 389–396

Vallian S, Gaken JA, Trayner ID, Gingold EB, Kouzarides T, Chang KS and Farzaneh F (1997) Transcriptional repression by the promyelocytic leukemia protein PML. *Exp Cell Res* **237**: 371–382

Wang S, Lee RJ, Cauchon G, Gorenstein DG and Low PS (1995) Delivery of antisense oligodeoxyribonucleotides against the human epidermal growth factor receptor into cultured KB cells with liposomes conjugated to folate via polyethylene glycol. *Proc Natl Acad Sci USA* **92**: 3318–3322

Wong AJ, Bigner SH, Kinzler KW, Hamilton SR and Vogelstein B (1987) Increased expression of the EGF receptor gene in malignant gliomas is invariably associated with gene amplification. *Proc Natl Acad Sci USA* **84**: 6899–6903

Yarden Y and Ullrich A (1988) Growth factor receptor tyrosine kinases. *Annu Rev Biochem* **57**: 443–478

*© 1999 Cancer Research Campaign*
